# TAI-PRM: trustworthy AI—project risk management framework towards Industry 5.0

**DOI:** 10.1007/s43681-023-00417-y

**Published:** 2024-02-14

**Authors:** Eduardo Vyhmeister, Gabriel G. Castane

**Affiliations:** Insight Centre of Data Analytics, University College Cork, Cork, Ireland

**Keywords:** Trustworthy AI, Artificial Intelligence, Risk Management, FMEA, Industry 5.0

## Abstract

Artificial Intelligence (AI) is increasingly being used in manufacturing to automate tasks and process data, leading to what has been termed Industry. 4.0. However, as we move towards Industry 5.0, there is a need to incorporate societal and human-centric dimensions into the development and deployment of AI software artefacts. This requires blending ethical considerations with existing practices and standards. To address this need, the TAI-PRM framework has been developed. It builds upon established methods, such as Failure Mode and Effect Analysis (FMEA) and the Industrial ISO 31000, to manage risks associated with AI artefacts in the manufacturing sector. The framework identifies ethical considerations as hazards that can impact system processes and sustainability and provides tools and metrics to manage these risks. To validate the framework, it was applied in an EU project for Digital Twins on AI for manufacturing. The results showed that TAI-PRM can effectively identify and track different failure modes associated with AI artefacts and help users to manage ethical risks associated with their deployment. By incorporating ethical considerations into risk management processes, the framework enables the developing and deploying trustworthy AI in the manufacturing sector.

**Supplementary Information:**

The online version contains supplementary material available at 10.1007/s43681-023-00417-y.

## Introduction

Artificial Intelligence (AI) has been instrumental in enabling Industry 4.0 to achieve its business objectives, with applications such as optimization, quality control, supply management, and generative design contributing to the industry’s competitiveness [32, 56, 58]. As a result, the Industry has seen increased production, greater customization, and improvements in maintenance, product, and process quality [6, 33].

Industry 5.0 builds upon Industry 4.0, incorporating ethical considerations for AI assets and placing humans at the core (human-centric), to address societal goals and support environmental and societal well-being through human–machine cooperation in smart working practices [4, 57].

Industry 5.0 adds new challenges such as real-time decision-making, human- centric solutions, edge computing, and transparency, which can be supported and driven by managing approaches and AI techniques [19]. The challenges of Industry 4.0, such as security, budget, talent, data analytics, integration, and procurement limitations, continue to be relevant [1, 17].

As with any new technology, trust in AI is perceived as a benefit, and the lack of it is seen as a risk to adoption and user acceptance [5]. Given that the actions of any agent over humans must be reliable, it follows that AI technology must be trustworthy [3]. This requires AI technology providers to minimize any risks related to its performance and impact on its users from a manufacturing perspective [10, 44, 45].

According to the high-level expert group set up by the European Commission [16], trustworthy AIs have three main pillars, which should be met throughout the system’s entire life cycle: it should be lawful, ethical and robust.

Trustworthy AI (TAI) is characterized by adherence to seven requirements: Human agency and oversight, technical robustness and safety, privacy and data governance, transparency, diversity, non-discrimination, and fairness, societal and environmental well-being, accountability. These requirements aim to ensure that AI systems respect fundamental rights, are secure and reliable, protect privacy and data, are transparent and explainable, avoid bias and promote stakeholder participation, promote societal and environmental well-being, and are subject to accountability mechanisms.

The goal of each requirement is to minimize the risks associated with AI and its potential adverse outcomes. Therefore, it is important to incorporate mechanisms to identify and manage these risks throughout the AI life cycle.

Furthermore, AI ethics and user acceptance are critical considerations in the development of AI systems. The principles of accountability, responsibility, and transparency (ART) have been identified as key drivers for responsible AI development [13]. These principles predate the European Union Trustworthy requirements and the European AI Act [9], and continue to be relevant in the development of strategies to promote safe and reliable human–AI interactions. However, incorporating these principles into management units can be challenging due to their ambiguity.

Ethical considerations can be incorporated into AI assets at different levels: ethic-in-design, ethic-by-design, and ethic-for-design. Ethic-in-design involves incorporating trustworthy concepts during the design phase. Ethic- by-design involves building components with intrinsic ethical capabilities. Ethics-for-design involves specifying ethical considerations within standards and regulations.

Human-centric AI is essential for Industry 5.0, emphasizing the collaboration between AI-driven systems and humans. This requires AI assets to be transparent, reliable, safe, and maintainable while considering human needs and experiences [12, 29]. The interaction between humans and AI in manufacturing can be categorized into three types (human-in-the-loop, human-on-the-loop, and human-in-command), depending on the level of human involvement. Importantly, the decisions made by these systems can be influenced by humans and data; thus, impacted by societal, legal, and physical considerations [12, 30, 54].

Several organizations have developed methods to facilitate practitioners implementing ethical principles in AI, including The Institute for Ethical AI and Machine Learning, Microsoft’s Responsible AI guidelines, UNI Global Union, the IEEE Global Initiative on Ethics of Autonomous and Intelligent Systems, and the ISO/IEC JTC 1 family focused on trustworthiness [20, 23, 24, 27, 35, 39].

In a 2020 study, Hagendorff presents a list of guidelines and strategies for addressing critical AI issues [21], pointing out what areas are making more attention. However, the study does not provide specific definitions for handling AI assets throughout their life cycle [15, 21]. Furthermore, the deployment of these approaches in different domains and environments can pose certain risks that need to be traced and understood [8, 12, 50].

The literature offers various approaches to integrating TAI considerations and risk management in the manufacturing sector. Developing a framework that ensures the ethical use of AI while minimizing potential harms and maximizing benefits requires a comprehensive approach that considers diverse stakeholders and technical aspects of risk management. So, how can a framework be implemented in the manufacturing sector considering approaches already in place? Frameworks that consider merging TAI with existing risk management approaches have already been considered. For example, Novelli et al. [41] integrates the AI Act with a risk assessment model inspired by the Intergovernmental Panel on Climate Change and related literature. The model aims to estimate AI risk magnitude by considering interactions among risk determinants, individual drivers, and multiple risk types.

ISO 31000 is widely used in the Industry as the main standard for risk management processes due to its versatility and suitability for various application areas and software life-cycle processes [22]. It describes procedures for assessing, treating, monitoring, reviewing, recording, and reporting risks. ISO 31000 is often applied with other more specific standards, such as ISO 9001 for supply chain and product quality improvement [42, 43, 47, 55], and ISO/AWI23247 for digital twin manufacturing framework [25]. However, there is a need to review and update these standards to meet the requirements of smart manufacturing and Industry 5.0 [38]. The applicability of different standards may lead to cross-violations depending on the context of their application, as they often use different methodologies [49].

We propose the TAI-PRM framework for process risk management to address this gap. This framework adds TAI requirements to existing risk management methods used in industrial processes, based on the ISO 31000 standard [26]. The TAI-PRM framework is designed to comply with current and future regulatory conditions on AI, and its objectives are to (1) Identify and evaluate risks associated with AI in the manufacturing sector, (2) Develop and implement risk mitigation strategies that align with TAI requirements, and (3) Monitor and review the effectiveness of risk management strategies over time.

By incorporating TAI requirements into risk management practices, the TAI-PRM framework can help ensure that AI is developed and used responsibly and ethically in the manufacturing sector.

The specific goals of the framework are:

- To support management units and developers in incorporating trust- worthy requirements within the AI lifecycle process.
- To secure the use of AI artefacts independently of legal and technical changes. The legislation heterogeneity applied in different countries on the use of AI can be varied; therefore, flexibility is key.
- To ease the combination of the frameworks that handle ethical-driven risks with other approaches commonly used for risk management. It needs to be designed as a complementary asset to these used by the Industry and not a replacement to facilitate its adoption.
- To facilitate an iterative process to handle risks on AI artefacts within the framework. Many processes in software do not follow sequential development but spiral/ iterative development processes. Therefore, the framework must flexibly adapt to any development cycle applied by developers for its incorporation.
- To ensure that Key Performance Indicators (KPIs) can be tracked through well-defined metrics that register the progress on the identified risks handling. Tracking KPIs is essential for their daily operations and business units. In addition, managerial levels can use these indicators to understand the impact of incorporating ethical aspects.
- To construct an architecture that addresses persona responsibilities and channels. With this aim, the framework must foster communication between technical and non-technical stakeholders.
- When possible, foster the reuse of outcomes in other research areas and market segments to avoid duplication of effort. This is translated as savings in revenue and research time on future developments. In addition, well-structured risk identification can avoid the repetition of failing conditions on AI components with similar target objectives.
- To enable a seamless path for the transition to Industry 5.0. By linking ethical considerations and risks, the Industry can handle TAI requirements as a risk management process (RMP).
- To provide users with a tool for performing the TAI-PRM and evaluate approaches already developed for TAI.

TAI-PRM has been implemented in a European project [2] that incorporates different manufacturing partners. By applying it, different failure modes have been identified as potential interests for TAI consideration. These results are shared within the mentioned tool, allowing expansion from those interested in its application.

TAI-PRM builds on two earlier works: the first one provides a scope contextualization that aids in comprehending the risk management process and evaluation [52]; the second establishes the groundwork for AI development and management, establishing connections between requirements and risk components [51]. These two works, together with the current one, can be used to define operationalization approaches for the framework proposed and facilitate the translation of the framework on domains different from the main one focused here (i.e. manufacturing) or possibly include other ethical- driven requirements within the framework. Furthermore, in the broader con- text of responsible AI development, it is essential to consider a systematic procedure for incorporating ethical considerations and different perspectives into the higher level of decision-making (e.g. risk management process it- self). Different principles can serve as a foundational starting point. For the case study defined, the ART principles approach [11]) was considered for implementation. This process involves engaging key stakeholders, documenting their values and requirements, aggregating interpretations systematically, and establishing links between values and norms (or rules to follow). The aim is to translate overarching ethical principles into concrete norms and functionalities, recognizing and addressing the unique aspects of each project. The implementation of this approach is out of the scope of this manuscript, which focuses on a framework implementation for assets management. Here is presented the framework after the full execution of the mentioned project and thus, consider all iterative processes within its development and implementation.

## Methodology

This section describes the FMEA (Failure Mode and Effects Analysis) methodology used in TAI-PRM and its relevance in AI assets. This section also justifies why the FMEA method was chosen as the risk assessment method. To integrate ethical considerations into risk management processes, the concept of ethical risks (e-risks) is introduced. E-risks are defined as conditions and processes that may have varying probabilities of materializing and can disrupt the expected behavior of an AI asset (and its use) due to the lack of consideration of TAI requirements, including values, social, legal, environmental, and other value-based constraints. For example, given the Technical Robustness Consideration from TAI, a risk could be a lack of human traits representability given that datasets do not include different accessories that can hinder human recognition. Other examples can include accountability and data governance considerations by merely the lack of policies to remove historical records, and vulnerable individuals or individuals about whom sensitive data is kept might be affected to a very high degree by inappropriate disclosure of personal data. In each case, if the risk materializes, there could be a disruption in the expected behaviour or use of the asset due to the value-based constraints.

The FEMA method is commonly used for risk assessment in various industries, including the technology sector. When it comes to assessing risks derived from TAI considerations, The FEMA method can be an effective tool for several reasons:

*Holistic approach*: The FEMA method considers potential failure modes and their effects on the entire system, rather than just focusing on individual components. This holistic approach aligns with the principles of TAI, which emphasizes the need for a comprehensive approach to ethical and technical considerations.

*Identifying potential hazards*: TAI requires identifying potential hazards, assessing their likelihood and impact, and taking steps to mitigate them. The FEMA method provides a structured framework for identifying and assessing potential failure modes, which can help uncover hidden hazards and ensure that all risks are considered.

*Systematic approach*: The FEMA method involves a systematic approach to risk assessment, including identifying failure modes, ranking them by severity, and developing mitigation strategies. This approach can help ensure that risks are addressed in a consistent and comprehensive manner, which is critical for TAI.

*Continuous improvement*: The FEMA method is designed to be an iterative process, with ongoing monitoring and evaluation of risks and mitigation strategies. This aligns with the principles of TAI, which emphasize the need for ongoing monitoring and continuous improvement to ensure that AI systems remain ethical, transparent, and accountable over time.

Overall, The FEMA method can be a valuable tool for assessing risks derived from TAI considerations. Its holistic approach, systematic method- ology, and emphasis on continuous improvement can help ensure that AI systems are designed and implemented in a responsible and ethical manner.

**Fig. 1:**
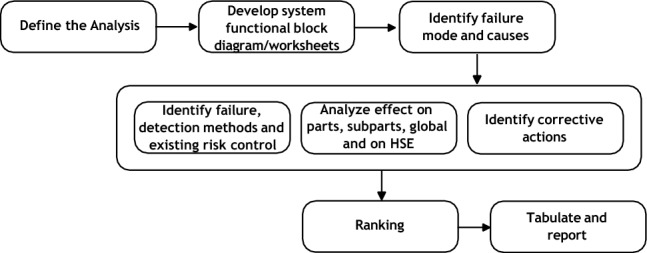
FMEA process

Figure 1 outlines a high-level view of the process. Key steps are explained next.

*Define the analysis* Developing TAI systems involves defining their constraints and potential failures, considering the system’s context and objectives, trustworthy requirements vary, such as the need for explainability in decision-making AI systems.

These considerations are outlined in the AI Act [9]. Nevertheless, these considerations need to be adapted to the specific industrial context and goals of the system—methods used to achieve it will depend on the system’s functionalities, data usage, user technical under- standing (and needs), and objectives.

*Development of system functional block diagrams*: Various sup porting documents, including functional block and reliability block diagrams, aid in analyzing potential failure modes and their impact. The level of de- tail should correspond to the risk level, with worksheets and cause-and-effect matrices being valuable tools. Additional information, such as system and AI boundary descriptions, design specifications, safety measures, safeguards, and control system details, should also be provided for effective risk analysis [7, 18].

*Identify failure modes* Identifying failure modes is crucial in understanding the underlying causes of system failures. This includes premature or spurious operation, intermittent operation, failure to operate and stop operation when required, loss of output, and degraded operational capability.

AI failure modes typically lack consideration of industrial context and specific trustworthy requirements. For example [31], define failure modes in IT safety, while FMEA has been applied to TAI, albeit with limited success in addressing only one requirement such as fairness [36].

While technical definitions of failure modes are well understood, non- technical perspectives can also consider implications such as the absence of supportive norms (action-guided rules), norm transgression, transgression to user’s values, and violation of corporate social responsibility [14].

We extend the scope of failure modes by incorporating 11 ethical-based failure families of functionalities derived from system norms and TAI requirements. These include failures related to robustness, safety, transparency, accountability, societal well-being, environmental well-being, human agency and oversight, privacy, data governance, bias (diversity, non-discrimination, and fairness), and users’ values. This differentiation facilitates the detection and definition of metrics.

Identifying failure modes involves measuring observable conditions or contingencies using supporting protocols that consider technical and non- technical operational modes. These conditions were grouped by drivers for failures such as physical, social, data, user/system interface, and algorithms. Our use case analyses confirmed the relevance of these drivers in identifying failure modes.

Physical drivers include power supply, communication/data link cables, robot parts, wearables, lenses, and sensors. Internal social drivers relate to stakeholders’ values and biases, such as enterprise social responsibilities and ethics-in-design. Data drivers encompass sources that could affect the trustworthiness of AI elements, such as biases, quality, quantity, and security. The user and system interface driver is linked to inadequate use of user interfaces and system inputs, including the absence of information display, tutorials, and guidelines, and the user’s malintent. Algorithm drivers include processes followed in problem-solving operations and expected functionality during the execution of AI code.

The 11 failure modes were tested by identifying the driver of the failure, which triggers a warning that identifies the failure mode and links it to an ethical-based general failure mode family. Further details on the failure modes are discussed in Subsection 5.1. A broad description of the failure modes tracked and found are included as contributions in the research performed.

The following three steps can be executed in parallel (see the middlebox in Fig. 1):

*Identity failure, detection methods and existing risk control* This phase involves detecting and managing failure modes using metrics and methods to reduce risk conditions. Adequate intervention procedures are needed for high-risk AI components. Linking further actions, such as control devices and circuit breakers, is crucial to enhance system understanding and reduce risk. Lack of detection methods could affect system robustness, security, and transparency. The importance of these actions should be linked to the intrinsic risk level of the AI assets involved.

*Analyze effects on parts, subparts, global, and on HSE (Health, Safety and Environment)* This step analyses the consequences of failure modes, including the end effect and its impact on social-driven components and each HSE element. A comprehensive analysis of AI interactions is required for complex architectures to identify any potential cascade effect.

*Identify corrective actions*: The third step involves identifying actions that can prevent or reduce the likelihood of failure, comply with legal requirements, ensure the safe operation of the system, recover from failures, and incorporate norms based on user values and AI trustworthiness requirements. These actions are contingency plans that are put in place in case a failure mode occurs.

*Ranking*: The ranking process assigns values to each failure mode’s likelihood, severity, and detectability, which can be used as KPIs for estimating the TAI state. The values are based on a soft metrics system and are used to prioritize actions to reduce the likelihood of failure, mitigate consequences, and improve detection.^1^

The Risk Priority Number index (RPN) concerning a concrete failure mode enables the normalization of the AI artefacts risks—Eq. 1. In Eq. 1 S is the severity (scale of 1–10, with 10 being the most severe), O the occurrence or likelihood (scale of 1–10, with 10 being the most likely), and D corresponds to the detection ranking (scale of 1–10, with 10 being the least capable). The RPN indicates the risk level of the failure mode, with a higher RPN indicating a higher risk for the AI component. The item or component is identified as a source of a failing condition with different impact levels based on the accumulated RPN.

1 $$RPN_{item} = S \cdot O \cdot D$$

To evaluate the overall risk of an AI component on different failure modes, the Global Risk Priority Number index (GRPN_item_) is calculated by summing the RPN of each item *i* to its corresponding failure mode ratio, as shown in Eq. 2*i* represents the different Failure Modes linked to the same source; n is the number of failure modes of a specific component; and α_i_ is the failure mode ratio and represents the part attributed to a concrete failure mode if the failure materializes. This means the percentage of the AI asset to fail with the specific failure mode.

2 $$GRPN_{item} = \mathop \sum \limits_{i = 0}^{n} RPN_{ii}$$

The failure mode ratio can be estimated as α_i_ = $\frac{ei}{etot}$. e_i_ is the amount of failing conditions of a specific failure mode, and e_tot_ is the total amount of all failing conditions in the system. The failure mode ratio was used to group risks with the same trustworthy requirement, thus identifying the most significant risk consideration for AI assets.

*Tabulate and report* The tabulation process for defining the overall risk level of AI components will be discussed in Sect. 5.2. The report includes documentation and a repository for users to understand failure modes, risks, control measures, safeguards, and recommendations. The risk register stores identified risks, along with risk management proposals, KPIs, evaluation methods, and contingency plan information.

## TAI-PRM: general considerations

This section covers necessary considerations before proceeding with the TAI-PRM. As a reminder, readers are encouraged to check previous publications in check approaches of extensions to other domains, regulatory conditions, or management approaches [51, 52].

The TAI-PRM adheres to the ISO3100, TAI considerations and requirements, and adopts a RASP approach, which includes a Risk Architecture, Strategy, and Protocols for risk management. We do not focus on discussing the validity of TAI, AI act, and ethical requirements given some specific do- mains (e.g. [34]), TAI-PRM focuses more on the operationalization of ethical considerations and thus provides a framework to perform such task. RASP provides a clear structure for communication and reporting of failures, strategies for implementation by individuals or organizations, and guidelines for managing risks. The first two components are discussed in [51, 52], while the focus of this work is on developing and testing the protocols for the risk management process. The following considerations were also defined: (i) the communication and consultation activities are part of the RASP architecture, not the risk management process itself; and (ii) the ethical risk management process includes the ISO-defined Monitoring and Review process as the primary component after Risk Evaluation and risk treatment.

AI assets were viewed as individual components that interact with other elements and can be embedded within systems or standalone. Dependencies of AI assets are classified in [52].

The illustrations use UML diagrams to represent the risk management process, with specific symbols to define activities. The diagrams show the starting point, activities, decision points, and endpoints. Some activities contain sub-flow charts, denoted by a [+] symbol.

**Fig. 2:**
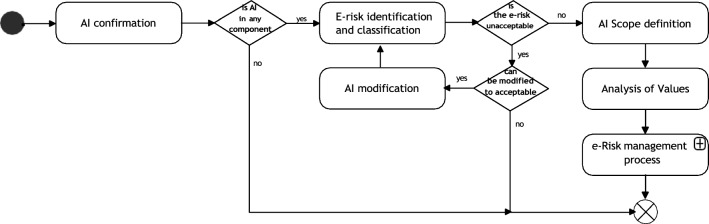
Benchmark e-risk management process

An overview of the TAI-RPM is shown in Fig. 2—more details at [51]. The Figure shows an abstraction of the risk management process with activities related to AI artefacts and TAI considerations. This flowchart should be driven continually and, as specified in our use case, the execution has been set when at least one of the following actions or states are identified:

- Initial AI design process.
- A new artefact is added to the system architecture.
- Modifications on a component with system dependencies.
- Changes on the data structure or its source.
- Incorporation of new functionalities to an AI artefact.
- Modification on interfaces and APIs.
- Changes on the context where the AI artefact is deployed.
- The regulations are modified or extended.

Figure 2 shows the first activity as AI confirmation, which involves identifying AI elements by defining and categorizing them.^2^

Next, the e-risk identification and classification activity focuses on identifying the AI elements’ intrinsic level of risk under regulatory conditions (i.e. given the current regulatory approach in EU, we focused on the risk levels defined by the AI act [9]). As previously claimed, to secure the use of AI artefacts independently of legal and technical changes. The legislation heterogeneity applied in different countries on the use of AI can be varied; therefore, flexibility is key. In this stage, and as explained in reference [52] a regulatory check process is included. Furthermore, since the approach proposed is fundamentally constructed for TAI requirements (with possible extensions, as covered in this manuscript), the possibility of extending the applicability of the framework within a similar environment of application should be possible.

If AI classification is deemed not acceptable, the user will proceed to either the AI Scope Definition activity or the <can be modified to acceptable?>  node. This node assesses whether the AI’s approach, data characteristics, and functionalities can be modified to meet TAI considerations for high-risk assets. If modification is feasible, the AI modification activity is executed to redefine the AI, followed by another round of <e-risk identification and classification> . If it is not possible to secure an AI asset acceptable risk level, the design or deployment process should be halted or decommissioned. In Analysis of Values (see [51, 52] for more information) is evaluated the potential for biases and use of personal information that could impact the requirements of Diversity, non-Discrimination, and Fairness (DnDF). It also considers the level of automation that is left to the AI asset and how this affects the agency of humans in decision-making processes.

The activity Analysis of Values allows integrating users’ values in the system. If there are conflicts, ANP or AHP tools can be used to resolve them for social, legal and ethical considerations. Finally, e-risk management process encapsulates the risk assessment, treatment, monitoring, and review- described in Sect. 4.

## e-risk management process

This section describes the risk management process, its context, and high- level activities. Thus it is connected with approaches thoroughly covered in previous references [51–53]. That cover general perspectives, how to establish the context of the risk management process, and how to merge the risk management process.

**Fig. 3:**
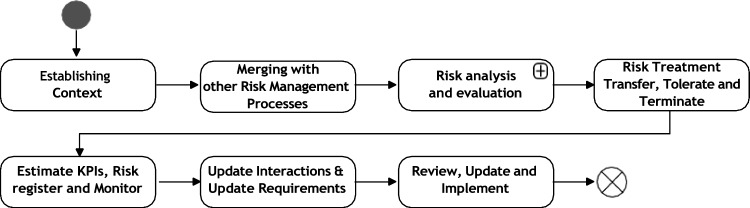
High-level overview of the e-risk management pipeline

**Fig. 4:**
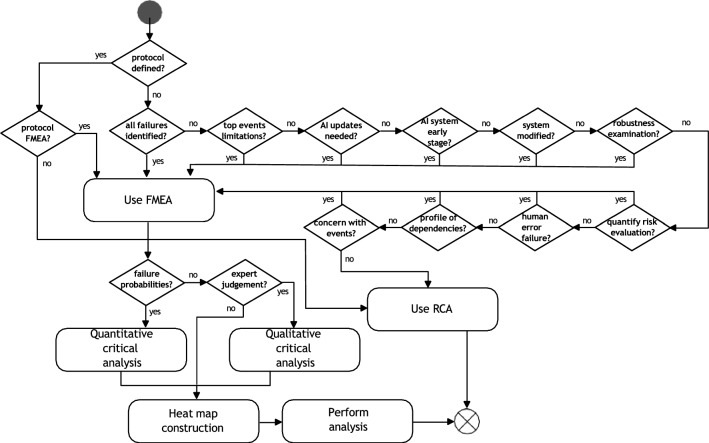
Risk analysis and evaluation flowchart overview

Figure 4 shows a high-level diagram of the ethical-driven risk management process.^3^ Each of the activities in the figure are discussed next (Fig. 3).

*Establishing context* In this activity, a gathering and run of information is performed. This is done by (1) defining how the AI asset interacts with other components and subsystems (i.e. software architecture documentation). (2) Define hierarchical extension of the components. Users must track cascade effects on risk analysis, for example using root cause analysis. (3) Analyze the interactions between AI elements actions, UIs, and humans, de- fined in the activity AI scope (Fig. 2 in Sect. 3). (4) Analyze constraints set up as requirements to control functionalities, input behaviors, system outcomes, and component values. If relevant, these constraints were established with the physical context, enhancing the system security, especially in AI-user interaction. (5) Analyze diagrams constructed on the information collected in Connectivity with other components and subsystems, Dependencies, and Human–AI and Human–UI interactions. (6) Analyze collected information from Requirements and values, as defined in Fig. 2 e-risk identification and Classification, AI Scope Definition[51, 52], and Analysis of Values.

*Merging with other risk management process* When several RMP instances are identified and similar approaches are used (i.e. FMEA) the analyses can be merged (e.g. merging with DFMEA and PFMEA processes; The DFMEA involves a comprehensive analysis of systems, while the PFMEA identifies and evaluates failures in processes).

*Risk analysis and evaluation* In this activity, the FMEA (and criticality analysis (CA) if information exists—FMECA) are used as core components for risk analysis. This is described in detail in Sect. 5 as it contains a detailed sub-flow’s core in the management of e-risks.

*Risk treatment, transfer, termination or tolerate* In this activity is evaluated, depending on the risk appetite and the risk levels, if it AI asset (or its responsibilities) should be treated (Failing conditions can be modified, upgraded, or safeguards), transferred (External safeguards will be allocating the responsibility of the failure events if materialized—Conditions can limit the option to transfer e-risks for TAI requirements), tolerated (No need of AI asset modification. Periodic updates on the status must be continued according to the frequency established in the risk management protocol) or terminated (the AI asset should not be used or developed). This activity is further extended in Subsection 6.

*Estimate KPIs, risk register and monitor* The activity of KPIs estimation is linked to trustworthy considerations of the risk management process, monitoring strategies, and protocols, failure modes, and the set- up of previous activities. The KPIs should be linked to each trustworthy requirement or value defined for AI. More details on the KPIs are provided in Section 7. In addition, the activity of Review, update, and implement involves integrating and keeping identified failure modes for posterior analyses from the same/similar system functionalities, interactions, or data usage. The monitoring part of the activity involves analysing and evaluating stakeholder outcomes to trace contingency actions, and the implementation of RMPs.

*Update interactions and update requirements* The outcomes of the previous process involve modifications related to AI assets and their interactions with other components, data structures managed by other AI assets, and the incorporation of additional AI assets or new functionalities that can impact the system’s trustworthiness. Therefore, it is necessary to analyse these new interactions to identify potential risks.

The iterative process of running the risk management must be tracked for accountability.^4^

*Review, update and implementation* Once there are no outstanding updates, the user must implement the risk treatment strategy, which depends on the interactions between the different personas involved in the risk management process as described in the Risk Architecture [51]. In addition, the status of the failure modes has to be revised, along with the mechanisms and processes to implement the 4 T’s—secure protocols, strategies, and control mechanisms for implementing AI assets. Internal or external auditing processes can be used to evaluate the system status and RMP. Finally, the risk register must be updated for accountability.

## Risk analysis and evaluation activity

In this section, it is detailed the activities for evaluating failure modes and how to address them. Thus this section covers crucial aspects of implementing FMEA or other risk management protocols.

Figure 4 extends the component described in the previous section. The figure depicts a flowchart that defines the instruments between the approaches FMEA, FMECA, and RCA (Root Cause Analyses) used for the risk assessment.

The first decision node, <Protocol defined?> analyses if the user has defined a FMEA or RCA protocol for risk assessment. This step is important to keep the integrity of the outcomes used for evaluation. If no protocol is defined, the set of decision nodes drives the user to the most convenient approach to follow. The set of questions are:

-  <All failures identified? > : This question addresses whether the user aims to identify”all possible” failing conditions. This means that the user is interested in detecting every situation that might trigger risk outcomes. Otherwise, failures can be unexpected.
-  <Top events limitations?> : This question focuses on the circumstance that the number of failure events is large or could be unexpected. Sim- ilar events sharing commonalities must be grouped under the same failure mode (e.g. two different fluid tanks could have the same FM if they run out of fluid).

-  <AI updates needed?> : When an AI asset requires human intervention or software updates, FMEA has extensive applicability and efficiency in managing this case.
-  <AI system early stage:?> This decision node addresses when the sys- tem is in the design or definition phase—A FMEA approach can better help to detect conditions that could lead to system failures; by analyzing pre-established failure modes of analogue systems, the process is facilitated.
- <System modified?> : This decision node analyses when the system will be modified considerably in future stages. Therefore several functionalities or interactions with other components might be modified as well.
- <Robustness examination?> : If AI artefacts are used in critical systems or failures can have severe impacts, this decision node will allow the user to decide which protocol to use. Furthermore, this also implies running Critical Analyses if the data can be linked to the probability of events of occurrences (More robust analyses).
- <Quantity risk evaluation? > : Since FMEA allows classification and grouping into failure modes in terms of their occurrence, severity, and detection, this decision node allows the user to decide the protocol.
- <Human error failure?> : This decision node addresses the systems that require considerable human intervention. The assignment of responsibilities can be facilitated, as well as the detection and control of the sources of risk. Nevertheless, care must be taken to draw clear limits between tasks activities and responsibilities.
- <Profile of dependencies?> : This decision node addresses when failures can trigger cascade events when they materialize. FMEA can foster a proper identification of system interdependence and perform better qualitative and quantitative analyses of cascade effects.
- <Concern with events?> : In this case, the user is asked whether an explanation of the relations between failures that can lead to severe consequences and impacts is required.

In case the user is led to Use FMEA activity—detailed in 5.1—, information regarding qualitative and quantitative evaluation must be gathered. Two decision nodes are used to define these <Failure probabilities?> and <Expert judgement?> . These led to the two activities in Figure Qualitative CA? and Quantitative CA?

Within them, CA could be performed. To performed, additional information such as the failure mode ratio (α), the conditional probability β, which represents the probability that the failure effect will result in the identified severity classification, and λ, which represents the overall system failure rate due to different causes over the operating time in units of time or cycles per time, is needed. Then, the Criticality Number (C_M_) provides a metric to classify a specific failure mode of an AI asset as follows (see Eq. 3):

3 $$C_{m} = \beta \alpha \lambda$$

The Overall Criticality Number (C_r_) estimates how critical an AI asset is with respect to a complete system—see Eq. 4:

4 $$C_{r} = \mathop \sum \limits_{i = 1}^{n} (C_{m} )_{i}$$

Furthermore, a heat map and a risk matrix must be constructed to keep the quantitative information tracked and to incorporate the numerical analysis based on the probabilistic information collected about the failure modes (as described in Sects. 5.2).

If the RCA is defined to be used, four different steps should be run: scope of RCA, root cause analysis, analysis/recommendations and record results. The application of the RCA methods is supported by different methodologies—the five why’s, change analysis/event analysis, or fishbone diagrams. The RCA is a well-documented approach beyond the scope of this paper, and therefore, it is not further explained.

### Use FMEA activity

**Fig. 5:**
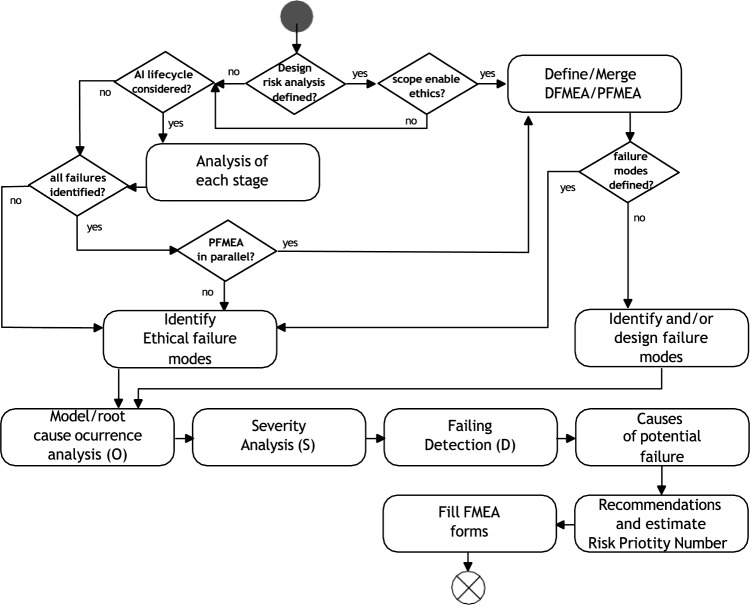
FMEA pipeline

Figure 5 shows the FMEA pipeline described in Fig. 4. The flowchart guides the user to merge the risks through other risk management approaches (if any), to identify the failure modes, and to determine the rates for the failure modes to perform post-analysis.

The process starts with a decision node, <design risk analysis defined?> , where the user can check if PFMEA or DFMEA protocols are run in parallel with TAI-PRM. When this occurs, the next decision node, <scope enable ethics?>  checks if the scope of the framework and the ongoing approaches can be merged or extended. This means to define components, items, dependencies, and to establish similitude between policies.

An activity named Define and Merge DFMEA/PFMEA defines the strategy to extend the functional blocks—if the process is running.

The decision node towards the left of the start – <AI lifecycle considered?>—checks whether the user has considered analysing the complete AI asset life cycle. If that is the case, an FMEA approach must be considered for each stage of the AI life cycle for the same asset within the Analysis of each stage activity, analysing the risks involved during each phase: design, development, use, and decommissioning.

The following decision node, <All failures identified?> , checks if the AI asset is used within maintenance or operational processes. If it does, a PFMEA process must be set for the combination of both processes. This must be executed by the user within <PFMEA in parallel?>.

Independently of the path that the user takes for the FMEA flow, TAI- PRM provides two activities key to identifying failures. The first, named Identify Ethical failure modes defines the scope of the analyses of ethical, trustworthy and values to be considered by the user. The second activity is identifying and/or design failure modes where, based on the design of the AI asset, the user must identify the system’s failure modes, subsystems, or components under the analysis of the present risk assessment.

A list of over 130 failure modes related to trustworthy considerations are identified,^5^ but are not limited to these. The failure modes described have the following structure: identifier, the driver, the family where they belong, a definition, and an example.

This list is based on a literature review and the industrial feedback from case studies in which the framework has been surveyed, focusing on developing solutions for the manufacturing Industry and using Artificial Intelligence to optimize production systems.

Furthermore, the tool constructed to perform TAI-PRM (described in the Sect. 8 of the document) further facilitates the identification of failure modes and adds the capability to incorporate and share Failure Modes between tool users.

After identifying the failure modes, a ranking activity of these must be executed.^6^ As observed in the Figure, for each failure condition—Model/Root—, on each component, the likelihood/occurrence rank (O), Severity (S), and Detection (D) must be considered. For the likelihood, confusion matrix analyses from the AI artefact can be linked to the probability of occurrence. If no probability numbers or historical information is accessible—this is quantitative information –, expert judgment can be used as an oracle.

When these analyses are performed, the user must consider that one or more Failure Modes can cause a failure condition and that it might be the case that a cascade effect is triggered, including PFMEA and DFMEA elements. This means that the effect can be local or global within the same or other subsystems.

It should be considered that if the detection (D) is performed by an entity external to the system, for example, throughout IoT devices, it should be considered the constraints of the communication channels. This could drive making the risk appetite more stringent under these circumstances.

The final three steps of the FMEA process focus on documentation for improving the detection and accountability of future failure modes. The first, Causes of potential failure, focuses on keeping control of the failure mode causes for future corrections. It is recommended to document these with a description of the cause following the rule” if X occurs, then Y happens” where X is the failure mode, and Y is the origin of the risk condition.

In the case of Recommendations and estimate the RPN activity, failure compensating previsions, functionality extensions or restrictions, or AI asset modifications should be documented to prevent/reduce the likelihood and severity, or improve the detection of the failure modes. Finally, in Fill FMEA forms, the user must fill the risk register.

Heat map construction.

The heat map—also referred in the literature as risk matrix—provides a mechanism to identify and compare AI artefacts and failure modes associated to the risks. This component is directly linked to a process of Fig. 4 with the same name.

Authors in [40] detail how to construct a risk matrix. Nevertheless, an extension should be made depending on the intrinsic risk level of the AI asset, as defined in the AI act.

The basis for the risk matrix is the risk definition, which is the combination of the severity of a risk when it materializes and its likelihood. To describe the risk, a classification can be used for severity and likelihood following qualitative descriptions and scales.^7^

To calculate each value on the matrix, the user must evaluate following a logic rule: IF likelihood is p AND severity of consequence is c THEN risk is r. Each value must be mapped into one of the four clusters—Tolerate, Transfer, Treat and Terminate (see Subsection 6).

When the user performs FMEA-only process, the analysis can be run by aggregating related metrics—see Eq. 2 as per Sect. 2. As result, the failure modes can be allocated on a matrix constructed based on the likelihood scale, the severity scale, and the risk score, by using a direct translation of the risk appetite.

Next example illustrates the process through four steps to construct the risk matrix using the FMEA in quartiles (i.e. use of the RPN to drive the construction of the heat map)^8^:

- Define how many percentiles will be used to cluster the information. In undecided cases, define based on the quartile, median, and third quartile (three points).
- Construct a rank matrix. This is built adding severity on the y-axis, occurrence on the bottom x-axis, and the detection rate on the upper x-axis. Then, intersection multiplications cover all possible RPN (see described in the repository).
- Use the percentiles derived from the risk appetite and the RPN range (from 0 to 1000) for the risk level cluster definition. In the case of quartiles, the Q1 values will be considered tolerable, and would not require any modification from the current condition.
- Connect the clusters with the 4 T’s. For example, the Q3 implies that 25% of the risk is above this value and, therefore any item there can be considered high risk and should be terminated. Anything item in the interquartile should be treated or transferred to be considered as risk condition. These limits can be replaced based on the percentiles used to construct the risk matrix and the risk appetite/policies.

To link the heat map with the AI Act, different risk appetites should be considered, depending on the intrinsic risk of the AI asset—i.e. the higher the risk of the AI artefact, the more stringent must be the risk appetite.

Tables 1 and 2 impose the contingency actions for the risks associated to the 4 T’s: Treat, Transfer, Terminate or Tolerate, described in detail in Sect. 4. The first of these tables is directly linked to the criticality analysis, while the second is linked to the RPN numbers—i.e. purely FMEA based approach, and explained in the methodology section.

**Table 1:** Risk score ranges in function of the intrinsic risk level

Risk level	Tolerate risk score range	Treat risk score range	Terminate risk score range
Unacceptable risk	–	–	1–25
High risk	1–5	6–20	21–25
Limited risk	1–10	11–25	–
Minimal risk	1–20	21–25	–

**Table 2:** RPN ranges in function of the intrinsic risk level

Risk level	Toleraterisk score range	Treat risk score range	Terminaterisk score range
Unacceptable risk	–	–	1–1000
High risk	1–200	201–800	801–1000
Limited risk	1–400	401–1000	–
Minimal risk	1–800	801–1000	–

The tables are described in function as the risk level of the AI asset. The ranges of values were defined as structured per quartiles (from lower to higher). This definition was done to set a risk appetite representation qualitatively. Nevertheless, further refinement is required as further knowledge is obtained from implementing the RMP in AI assets and our use cases. Importantly, users can modify the proposed ranges based on the risk policies established in their own enterprises, always considering the constraints of the AI act.

## Risk treatment transfer terminate or tolerate activity

In this section, it is explained how to treat, tolerate, or terminate an AI process or asset based on risk appetite. The appetite should be considered as an aspect derived from the RASP approach and thus were encouraged to review previous publications.

**Fig. 6:**
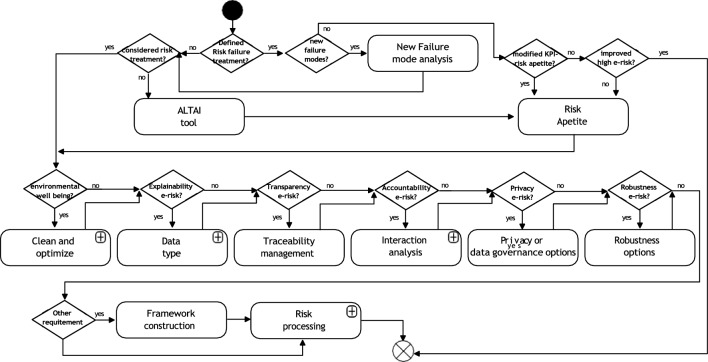
4 T’s—Risk treatment, transfer, terminate or tolerate flowchart overview

Figure 6 shows the flowchart associated with the 4 T’s of risk management, which is the fourth step of Fig. 4. The first decision node —<Define Risk failure treatment?> —points to the user if has previously been run or if this is a new RMP process. If so (i.e. a repeated RMP) the user is driven to a decision node named <New Failure modes? >  that established if new failure modes have been identified for the AI asset. If so, it is required to perform a new evaluation of the failure mode under the 4 T’s analyses (i.e. previous section).

When the user does not identify new failure modes, then the decision node <modified KPI risk appetite?> checks if there were modifications to the risk appetite. If positive, additional KPIs are considered for the risk management process. If negative, the user must evaluate <improved high e- risk?> . In this case, the question guides the user to understand if e-risks have been addressed, improving their conditions under the risk evaluation. If no further system modifications are allowed/needed and KPI are accepted, the process ends. Otherwise, before executing the activity risk appetite, previous historical records must be analysed to understand improvements.

The activity risk appetite checks if modifications are needed on the risk appetite based on policies established for the risk management process. This means setting up more stringent conditions for the risks under consideration or evaluating alternatives to manage the identified risks. This activity, once finished, should restart the 4 T’s analysis before continuing with the following stages.

In the case the process is a new RMP, the decision node <Define Risk failure treatment?> drives to the question <Considered risk treatment?> where the user must consider if recommendations and causes have been pre- established for the system. If not, the user must run the Assessment List for Trustworthy Artificial Intelligence (ALTAI) tool^9^ and evaluate the TAI guidelines.

Following a piloting process by the European Commission, the ALTAI tool was implemented. The tool performs a checklist that guides developers and deployers of AI in implementing TAI principles in practice. Regretfully, the tool describes an oversimplification of the ethical considerations and does not facilitate its implementation. Furthermore, given its disconnection to the AI act and risk management approaches, does not provide insight into imminent failing conditions, technical approaches of implementation, nor a continual approach of improvement.

The ALTAI tool activity seeks to extract considerations not foreseen by the current framework that can further contribute to the user with approaches to reduce the e-risks of specific Failure Modes.

The 4 T’s analysis comprises the bottom-central part of the Figure, with six decision nodes that encompass questions used to define recommendations to be implemented on the AI artefacts, depending if is defined to treat the AI asset and are recognized from the E-risk identification and classification step (see Fig. 2), the main components from the TAI requirements, that need further attention.

<Environmental well-being > checks if the TAI requirement, of the same name, needs to be evaluated for the AI asset. In addition, users can be considered to evaluate this activity if their interest involves sustainable approaches, protecting natural resources, and eliminating excessive energy usage (including training and optimization processes). The pipeline for this process, clean and optimize, involves four consecutive activities as follows:

Time frames process, considering the modification and update of processes timeframes. This involves—e.g. update of metaparameters, and avoiding continual training as long as the process robustness is secured; reducing data time stamps for analyses on processes that describe slow dynamics. Meta parameters and re-estimation activity is where the user must consider to secure the saving of runs results in highly energy-intensive processes such as optimization and to provide a method to reuse the results; Dimensionality process is where the user has to perform system dimensionality analyses to reduce the amount of data to be processed if justified—in additon to improve generalization. In this way it is avoided to perform analyses and iterative tasks on variables that are statistically the same, and to suppress recording unnecessary data; and Novel process incorporation of methods and adequate testing techniques to secure the reduction of the computational burden—e.g. metamorphic or regression testing. In addition, this implies the incorporation of more efficient processes compared to legacy ones.

**Fig. 7:**
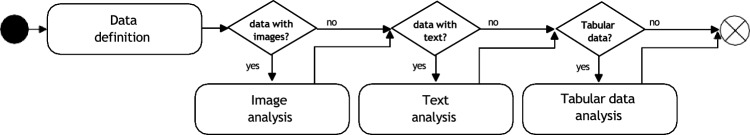
4 T’s—data type

Following with Fig. 6, the decision node <Explainibility e-risk?> evaluates if the explainability component of the transparency requirement of AI assets needs attention (again, as defined in E-risk identification and classification—Fig. 2). If the user has to perform this analysis, the decision node drives to the activity Data type where a distinction between data formats—images, textual, or tabular — must be taken. Figure 7 illustrates the sub-flow of the activity.

Once the data format is specified, suitable approaches for the system explainability can be used. Figure 7 follows this idea. If data corresponds to the image, the activity Image Analysis promotes the use of saliency maps, including a Digital-Twins-approach that could drive explainability over sys- tem; concept attribute, counterfactual, or prototypes. Importantly, natural language, depending on the approaches used, can use similar tools as Image analyses. If data corresponds to text, the text analysis: sentence highlight, or attention-based method can be used. Finally, if data is tabular, the activity Tabular data analysis promotes the use of feature importance—including digital twins, rule-based, prototypes, and counterfactual approaches. The TAI-PRM framework does not seek to constrain the user preferences on the algorithms used on the AI artefacts. However, specific cases can require detailed processes [28, 37, 48].

Following with Fig. 6, The decision node <Transparency e-risk?> focuses on the TAI requirement of traceability. Thus, the activity focuses on recommendations that define: (1) adequate documentation and protocols to keep records of data provenance, requirement, architecture, codes, minutes, and different text used to specify AI functionalities and updates (2) performance reports that can trace periodical evaluation of the system performance (KPIs)—defined in following sections.

Importantly other approaches, such as MLOPs have been reported to improve transparency and other trustworthy requirements. MLOps is a collaborative process between dev-ops engineers (software development (Dev) and IT operations (Ops)) and data science. As seen in the literature, few references are starting to focus on MLOps. Its use could enhance accountability and transparency of the processes involved and, given a proper definition of processes, produce an extension to different AI.

The <Accountability e-risk?> decision node is activated if accountability requirements are set to the AI asset if it does, the activity Interaction analysis takes place. Here, three consecutive activities are performed. The first activity, named Responsibilities and Obligations, establishes responsibilities for AI artefacts under failing conditions. The AI asset’s responsibilities and obligations must be established under failing conditions. Developers are responsible for design faults, while users are responsible for faults resulting from design requirements and operation. Users must set conditions where accountability is not assigned to users or designers for unforeseen interactions. Regardless of accountability, clear obligations and contingencies must be established based on the risk assessment.

To facilitate the definitions of responsibilities and obligations, protocols and approaches must be established to secure accountability. This involves defining protocols for reporting failure conditions, monitoring anomalies in data and processes, auditing strategies, and certification processes that foster transparency in the system.

The second activity, named human-in-the-loop, human-in-control, and human-on-the-loop seeks to expand the human agency and oversight TAI requirement by defining, depending on the level of responsibility of the user’s, accountabilities among the AI asset and the user. These considerations should be defined based on: how much autonomy should be given to the AI elements. This question implies that general frameworks should secure the incorporation of human agency in the manufacturing sector by considering the use of human-in-the-loop (HITL) human-on-the-loop (HOTL) or human-in-command (HIC). The choice of which approach would be more suitable depends on the complexity of the decision to be made and the user’s level of expertise involved in the decision-making process. In producing goods with embedded AI elements, a continual agreement with the end-user should be embedded to supply enough information to make its own decision. Pre- stated options should be avoided, given human bias tendencies to consider those machine-based decisions are more suitable than pure human-based approaches.

The final activity is named Interfaces analysis. Users must specify the type of interaction with interactive AI artefacts, thus setting, again, the responsibilities of users when interacting with the AI asset. Further approaches could be implemented to secure the minimization of negative impact and re- dress.

The decision node <Privacy e-risk?> , form Fig. 6, focuses on whether privacy is needed and if the user identified or understands this aspect of the AI asset. If necessary, the activity Privacy and data governance options is executed and includes the following steps:

To comply with GDPR regulations, take steps such as avoiding sensitive data collection, minimizing data retention time, ensuring IT security protocols, training for data processing, assessing data portability, informing users, providing a contact point for privacy concerns, and selecting experienced data processors.

The decision node <Robustness e-risk?> points to the user to understand the system’s needs concerning the recovery of failures. The recommendations produced from the activity Robustness options include to: (1) specify the system performance metrics, (2) perform functional testing, (3) check performance through benchmarking, (4) test the development using simulation, (5) set up an environment for anomaly monitoring, (6) set fail-safe mechanisms, (7) set hardware security, (8) define data quality/quantity, and (9) define improvements.

Next decision node, <Other requirement?> asks whether the user specified any other requirement that needs attention. If positive, the Framework Construction activity settles extensions to the TAI-PRM to handle new approaches, mechanisms, protocols, or new requirements continually. This activity must be compliant with regulatory conditions.

Finally, the activity Risk processing addresses the 4 T’s alternatives to manage risks. It involves three separated and consecutive activities risk treatment, Risk terminate, and Risk transfer.

Depending on the risk appetite, the policies from the management group, the results obtained from the FMEA analysis, and the recommendations established on the Risk analysis and evaluation process—Fig. 5, the processes of Terminate, Tolerate or Treat the AI assets should be established.

These stages refer to summarize all previous analyses to define final conclusions and actions to be taken based on recommendations over the AI assets. Independent of the action taken, it is recommended to secure the mechanisms to keep them evaluated by the corresponding KPIs to check their status in the risk register.

## Metrics

This section covers the metrics associated with evaluating risks and the framework and perform continual improvement on the assets under consideration.

The fifth stage of Fig. 4 focuses on possible KPIs for managing AI as- sets and their e-risks. These metrics are (1) associated with FMEA and FMECA, (2) TAI-PRM ethical-based, (3) TAI-PRM independent, and (4) environmental, social, and governance metrics. A nomenclature table is included as supplementary information if needed.

### Metrics for FMEA and FMECA

These metrics can be used to trace the risk tendency when FMEA / FMECA are used. They could be grouped by specific risks family or requirement to define the most conflicting (and therefore the highest attention needed) requirement for the AI asset(s). Table 3 describes these metrics. This table does not include those previously defined for FMEA (i.e. RPN, S, O, and D).

**Table 3:** FMEA and FMECA based metrics

Name	Definition
Item criticality number	Accumulated critical numbers over the same system. This accumulation could be driven by failure modes of different nature (e.g. Human agency and Over-sight vs accountability). The same scaling system should be used if accumu- lated over all j possible sources of critical numbers. *C*_*r*_ = $\sum_{{\text{n}}=1}^{{\text{j}}}{{\text{c}}}_{{\text{m}},{\text{i}}}$
Ethical critical number (ECN)	Criticality number for a specific trustworthy requirement and AI artefact. ${\text{ECN}}=\sum_{{\text{n}}=1}^{{\text{k}}}{{\text{c}}}_{{\text{r}},{\text{i}}}$
Ethical relative criticality number (ERCN)	Ratio of item criticality number a specific trustworthy requirement and AI artefact over the total critical numbers produced by the system: ${\text{ERCN}}={\text{C}}\_({\text{r}},{\text{i}})/ {\sum }_{{\text{i}}=1}^{{\text{n}}}{{\text{jC}}}_{{\text{r}},{\text{i}}}$ where k is previously defined and j is the scale

### Framework general and ethical-based metrics

These metrics complement the decision-making processes of the AI assets. Most KPIs should be associated with these metrics associated with the ratio values based on the risk limits. Table 4 describes these metrics.

**Table 4:** High-level metrics for management of risk ratio at overall system level

Name	Definition
%_LU_—unacceptable likelihood risk ratio	The likelihood of risks to materialize with an intrinsic value higher than the chosen by the management. It is calculated as: %_LU_ = N_i>LU_ /N_risk_ where N_i>LU_ is the number of risk with likelihood over the stated limits and N_risk_ is the failure modes identified
%_LA_—acceptance likelihood risk ratio	1—%_LU_
%_SU_—unacceptable severity risk ratio	Number of risks with severity over limit chosen by the management Number. It is calculated as: %_SU_ = N_i>SU_ /N_risk_ where N_i>SU_ is the number of risk with severity over the stated limits and N_risk_ is previously defined
%_SA_—acceptable severity risk ratio	1—%_SU_
DC—detection capacity	Average detection capacity of failure modes with risk levels over those in the stated limits. This number describes the average of how the system fails. It is calculated as: $\overline{{\text{DC}} }= \sum {{\text{RPN}}}_{{\text{i}}>{\text{DU}}}/{{\text{N}}}_{{\text{risk}}}$ where RPN_i>DU_ *i* = 1 is the Risk Priority Number with detection over the stated limits, *k* are all the risk over the stated limit and N_risk_ is previously defined

### TAI-PRM independent

These metrics are proposed to track the AI assets’ based on already- in-use metrics in the Computer Science domain or in the implementation domain (i.e. manufacturing). Some of these include, among others, True Positives (TP), True Negatives (TN), False Positives (FP), False Negatives (FN), accuracy (TP + TN)/(TP + TN + FP + FN), Error rate (FP + FN)/(FP + FN + FP + FN), Precision TP/(TP + FP), and F1-Score 2TP/(2TP + FP + FN) (Table 5).

**Table 5:** Metrics for AI components for software engineering non-ethical based

Name	Definition
AIEC AI effective capacity	Asset up-time from deployment (not training if applicable). If the use of the AI asset is discrete, it refers to the number of uses. When it is a DSS it must be considered the outcomes accepted by users with respect to the times it runs. It is calculated as: *t*_used_/*t*_total_ for discrete AI utilisation; t_up−time_ for continuous time
AIPPM AI planned maintenance	Ratio of time or cycles used per scheduled downtime operations. It includes training/parametrization and AI maintenance. *t*_scheduled_/*t*_total_
AIDR AI downtime rate	Ratio of unscheduled downtime. It must consider the unexpected events when the AI is idle or offline. This metric provides insights into AI stability *t*unscheduled/*t*total
AICU AI capacity utilisation	Amount of time when the AI should be utilised with respect to the total avail- able. The metric estimation is similar to AIEC with the difference that it considers only the functional time. *t*_used_/(*t*_total_ − *t*_scheduled_) for discrete AI utilisation; *t*_up−time_/(*t*_total_ − *t*_scheduled_) for continuous time
AICI AI correction indicator	Efficiency of the AI asset with respect two a KPI (KPI) to perform contingency actions before they occur, compared to previous information available on the system (KPI_initial_ − KPI_new_)/(KPI_initial_ − KPI_old_)

### Environmental, social, and governance metrics

These metrics are based on a high-level analysis of environmental, social, and governance metrics. Thus, they are not necessarily suitable for tracking internal risk management processes associated with AI artefacts.

Environmental, social, and governance (ESG) metrics are designed for investment analysis and may not be suitable for tracking internal risk management processes related to AI. ESG encompasses three areas: ESG integration, values-based investing, and impact investing. One example of an ESG rating is the Morgan Stanley Capital International ESG Rating,^10^ which measures a company’s resilience to long-term ESG risks using a rules-based methodology. The rating covers various types of securities, funds, and countries.

## Validation and running example

This section discusses the validation process and tools for implementing TAI-PRM and describes, in a summarized way as an exemplification approach, the findings when applying the method within a specific asset of a system constituted of several AI assets.

The TAI-PRM validation process was executed on multiple AI assets as a cooperation between different manufacturing companies and research institutions involved in the ASSISTANT project [2, 51, 52]. The projects secure the involvement of five academic and seven industrial partners with core activities on manufacturing. The environment has been used to develop and refine the framework with their inputs over multiple iterations. It is currently in use for its improvement.

Furthermore, a tool was developed to facilitate the implementation of the framework and evaluate the ALTAI tool and its applicability in the manufacturing sector.

The rest of this section describes the Assistant scenario as a case study, describe the use of TAI-PRM starting from Sect. 3, and further detail the tool that can be used and assessed by readers.

### Scenario overview

**Fig. 8:**
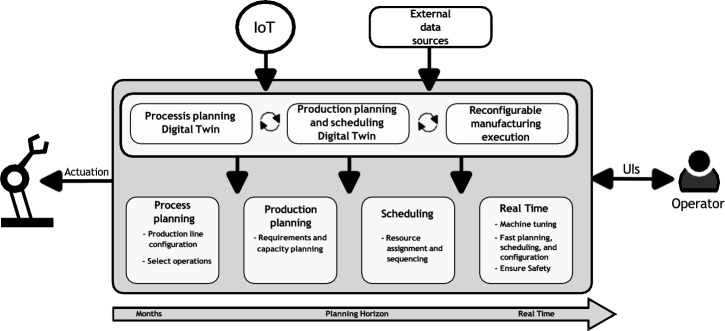
Set of interrelated twins for decision aid

ASSISTANT focuses on factories with production processes and assembly lines where multiple product variants are processed. Such production systems are composed of multiple stations, where the resources (workers/robots/tools) can move from one station to another. Using a set of digital twins and based on the data collected from IoT devices and external data sources (see Fig. 8), production planners and engineers will operate the manufacturing production through well-defined UIs.

Figure 8 shows the data are perceived from sensors in the factory and external sources, creating a real-time digital image of the factory. The reasoning step includes the decision models (process planning/production planning/scheduling/real time) built from the factory’s image and prescriptive analytics to create plans to be validated through simulation. The plan is executed through a real-time control module. The arrows in the figure represent the data flow/actions, whereas the circular arrows illustrate the feedback loops between the twins.

Three main blocks configure the ASSISTANT architecture. AI assets within them are required to be identified to proceed with the TAI-PRM. We used a taxonomic reference of the AI assets to understand the involved assets’ scope fully. we used the AI Watch taxonomy [46] for this task.

The main systems identified in the architecture are: (1) process planning, (2) production planning and scheduling, and (3) real-time control and actuation. The full description of these tables can be found in Supplementary Material.

### TAI-PRM applied to an ASSISTANT AI asset

The running example is focused on the asset Human Body Detection and Human Task Prediction Artefact defined in the supplementary, within the component Real-Time Control and Actuation. Following subsection describes action taken based on the pipeline processes presented.

#### Benchmark e-risk management process: an example

The first activity is identifying whether the component has an AI. By a proper analysis and following taxonomy, the AI artefact has three main functionalities (1) perception/computer vision, (2) Learning / ML, and (3) interaction/robotics and automation. Then the decision node branch chosen in is AI in any component? on Fig. 2 is YES.

The process of e-risk identification and classification is executed as de- scribed at [51, 52]. The AI asset has Limited intrinsic risk for (1) and (2) and, as evaluated within the process, a High Risk for (3). In the diagram, the decision node branch for < is the e-risk unacceptable > is NO. Based on the analysis, the requirements to be analyzed for functionalities (1) and (2) are: transparency, technical robustness, safety, and societal and environmental Well-Being. Functionality (3) must also consider accountability and human Agency and oversight requirements. Furthermore, the activity Analysis of Values does not produce additional requirements, as defined by industrial partners.

#### e-risk management process— example

Next, the activity e-risk management process is executed—Fig. 4. To facilitate the analyses, from here and forward, we will focus only on the perception and computer vision (functionality 1) of the AI asset.

The Establishing Context step was performed by analyzing the full requirements of the real-time control and actuation component. The connectivity with other components, subsystems, dependencies, and constraints, among other definitions, were defined.^11^

Since no further risk management processes were run in parallel in the ASSISTANT project, there were no considerations of merging TAI-PRM with other approaches—second activity in Fig. 4. The risk analysis and evaluation process (Fig. 4) was followed, producing the following flowchart outcomes associated with the decision nodes: <Protocol Defined?>: NO – <All failures identified>: NO – < Top events limitation > : NO – <AI updates needed> : NO – < AI system stage? > : Yes. Based on this information, the method used to perform the risk analyses is based on the FMEA approach *(i.e. Use FMEA).*

As a reference, Fig. 5 details the FMEA activity. The following decisions were defined by following the mentioned pipeline: < Design risk analysis defined > : NO; < AI lifecycle considered > : NO; < All failures identified > : NO. This leads to the activity Identify Ethical Failure Modes. The Table described in the repository was used to identify the different failure modes. Table 6 shows the failure modes detected based on the family (column Failure) and the name of the failure mode (Category column). The same table also includes the activity of ranking the Failure Modes based on the Occurrence (O), Severity (S) and Detection (D) of the failure conditions. The ranking was based on the tables included in the repository.

**Table 6:** Failure modes identified for the AI artefact under analysis

Failure	Category	O	S	D	RPN
Robustness	Poor human traits representability	4	5	4	80
	Disruptive traits	2	5	5	50
	Improper software use	3	5	5	75
	Interface handling	1	5	5	25
Transparency	Lack of explainable protocols	5	10	2	100
	Lack of documentation	10	4	5	200
Safety	Lack of contingency plan	1	10	5	50
Societal and environmental well-being	N.A	**–**	**–**	**–**	**–**

In the table, each RPN was calculated as the product of OxSxD. This is, for example, in the case of the failure robustness, and the category disruptive traits, 2 × 5 × 5 = 50.

The Societal and Environmental Well-being Requirements were set to be analyzed, however, no specific Failure Modes were identified for the AI artefacts and therefore, no further analyses were required.

The specifications were based on expert knowledge from industrial definitions and system developers. Further considerations, such as establishing protocols for risk management and policies from industrial partners, were established on the AI artefacts generated within ASSISTANT and considered in the definition of Failing Detection activity.

The activities—Causes of potential failure mode, Recommendations and estimate RPN, and Fill the FMEA form were run with no further actions required. The RPN were reported in the previous activities with no recommendations concerning the risk management process—their values were lower than the limits set for treatment.

Once the Use FMEA activity was finalized (4), the decision node < Failure probability? > reminds the user to collect information about probability and failure rates to perform a quantitative CA. Since no information was available, as it is an early phase of development for the artefact and no historical records are available, the output of the decision node is NO.

Alternatively, we used the RPN information for processing heat maps and performing decisions over the AI assets. Furthermore, the RPN was aggregated for the same artefact (Human Body detection and human task prediction—hbd in short) following two considering equal ratios of failures as follows:

$$\begin{aligned} GRPN_{hbd,robustness} & = 80*0.25 + 50*0.25 + 75*0.25 + 25*0.25 \\ & = 57.5GRPN_{hbd,transparency} = 100*0.5 + 200*0.5 \\ & = 150GRPN_{hbd,safety} = 50*1 = 50 \\ \end{aligned}$$

The GRPN_hbd_ is the addition of GRPN_hbd,robustness_, GRPN_hbd,transparency_, and GRPN_hbd,safety_ with a value of 257.5. The values for the RPN of each trustworthy requirement are equally weighted due to the lack of documentation that allows for pondering them accurately. However, these can be flexibly adapted depending on the scenario, where the asset will be operational.

Once the GPRN_hbd_ was obtained, it can be analysed against the heat map/critical matrix that corresponds to an element with Limited Risk—with respect to the classification of the AI component defined during the initial scope definitions activity. As observed in Table 2 considering our current risk appetite, a RPN of 257.5 corresponds to a “−” Tolerate” status of the AI component. This risk appetite could be more stringent depending on the risk policies established by the risk management group.

Once the Perform analysis process activity finishes—Fig. 4—the e-risk management process continues through Fig. 4. The risk treatment, transfer, terminate, or tolerate activity takes place.

Based on the previous analysis, the flowchart in supplementary material is used (together with the ALTAI tool) to identify possible modifications to the AI assets. After using the ALTAI tool, the recommendations, based on specific trustworthy requirements, are not needed. This is given since the asset does not contemplate treat. Furthermore, since there is no need to expand the framework, the last two activities Framework construction and Risk processing, do not perform any modification on the AI artefact, and the flow ends.

### TAI-PRM tool

A tool based on TAI-PRM has been developed for the ASSISTANT use case. The tool is accessible via the https://assistant.insight-centre.org/ page and offers comprehensive information on the risk management process. It includes two main sections with user feedback. Users can access information on how to perform the ALTAI tool or the TAI-PRM process. The tool is not an alternative to the ALTAI tool but rather helps to record the linkage and usefulness of trustworthy requirements, component assessment, and AI implementation functionality. The records will be compared to provide users with trends based on their domain. Users only interested in the TAI-PRM process need not complete the ALTAI component.

To use the tool, select the My TAI-PRM tab and create as many risk management processes as needed. Complete the pipeline before generating a downloadable report. The tool also allows users to create and share failure modes to extend knowledge based on the presented strategies. Users can provide feedback at the end of each TAI-PRM to improve the tool.

A total of 8 steps are needed to perform the TAI-PRM including: (1) Initiation of risk management process, (2) E-risk identification and classification, (3) AI Scope Definition and Analysis of Values, (4) Establishing Context, Merging with other RMP, and defining CA or FMEA, (5) FMEA or CA, (6) Ranking, (7) risk register, and (8) Treat, Terminate, Tolerate, and Transfer.

The tools follow a distribution similar to the ALTAI tool to familiarize users with both elements. Facilitating the acceptance of users that have already driven them.

Finally, information from the analogue of the ALTAI tool can be linked to a specific TAI-PRM, so information generated from it is also used in the final report of the TAI-PRM.

## Conclusion

This document presents a framework to develop and design AI artefacts in the manufacturing sector under responsible AI scrutiny. TAI-RMP proposes a well-structured approach based on risk management, including ethical concerns for AI artefacts. It extends the risk assessment methodology with TAI guidelines using FMEA. Further, a methodology is presented to define risk appetites and strategies in the industrial sector, to implement risk assessments under the intrinsic risk level of the AI artefact—and based on the Artificial Intelligence Act. Furthermore, the methodology is also blended with the 4 T’s on risk management: Treatment, Transfer, Tolerate or Terminate.

The framework underwent validation with academic and industrial organizations that improved its risk assessment, evaluation, and metrics. However, the framework’s application is limited to manufacturing, and additional definitions are necessary to create a comprehensive approach for managing AI assets under risk assessment. This includes evaluating and expanding the failure modes that could impact AI assets in various domains.

TAI-PRM can be used to implement trustworthy considerations and man- age risks in compliance with current regulations. It can also serve as a guide.

for developing standards for managing risks from AI assets. Feedback from various domains highlighted the importance of considering the human factor in risk management processes. However, the lack of regulatory conditions, certification, and standards for managing AI hinders adoption and implementation of transparent and accountable AI systems. Addressing this requires considerable training and effort to change business and development team operations.

We claimed that the framework helps in identification and tracking of failure modes (related to e-risks), help users to manage ethical risks, and connection with TAI based only on the case study provided (Not the whole project in which the work was applied), these contribution claims are as follows.

- *Identification and tracking of failure modes*: The case study describes the application of TAI-PRM to the ASSISTANT project, focusing on the Human Body Detection and Human Task Prediction Artefact within the Real-Time Control and Actuation component. The FMEA process is employed to systematically identify potential failure modes associated with the AI asset. The identified failure modes are categorized, ranked based on severity, occurrence, and detectability, and then aggregated to provide an overall RPN. The RPN is further analyzed using a heat map/critical matrix, providing a clear visualization of the risk level associated with the AI asset.
- *Helping users manage ethical risks*: TAI-PRM incorporates the concept of “trustworthy AI,” which includes ethical considerations. The framework defines trustworthy requirements that are used to assess and manage ethical risks. The case study explicitly mentions the consideration of ethical aspects such as transparency, technical robust- ness, safety, and societal and environmental well-being. The framework guides users through the process of analyzing failure modes in terms of their impact on these ethical dimensions. Recommendations and risk management decisions are made based on the analysis, allowing users to address and mitigate ethical concerns associated with the AI asset.
- *Connection with TAI*: TAI-PRM promises a systematic and comprehensive approach to AI risk management, considering both technical and ethical dimensions. The case study demonstrates the application of TAI-PRM in a real-world scenario, showcasing its effectiveness in identifying, assessing, and managing risks associated with a specific AI asset used in the manufacturing sector. The claim is substantiated by the detailed steps and activities performed in the case study, showing how TAI-PRM provides a structured process for handling ethical risks throughout the AI development lifecycle.

In summary, ethical imperatives, along with standards and frameworks, can drive the development of new AI assets for various industries. The ethical imperatives discussed in this work are related to the Risk Protocols of the RASP approach. Future work will gather information from multiple industries, covering detailed KPIs and extended metrics that will facilitate the incorporation of trust in AI artefacts for different areas such as healthcare and media, beyond manufacturing.

## Supplementary Information

Below is the link to the electronic supplementary material.

Supplementary file1 (DOCX 23 KB)

Supplementary file2 (DOCX 36 KB)

Supplementary file3 (DOCX 44 KB)

## Footnotes

1 More information and tables for the ranking process are available on a Github page https://github.com/lebriag/TAI-PRM/tree/main/Support/RPN%20ranking% 20tables.

2 The recommended categorization approach is based on the AI Watch definitions for European countries—https://publications.jrc.ec.europa.eu/repository/handle/JRC126426

3 Full detailed diagram at:https://github.com/lebriag/TAI-PRM/tree/main/ Flowcharts/e-risk%20management.

4 Extended diagrams available at:https://github.com/lebriag/TAI-PRM/tree/ main/Flowcharts/e-risk%20management/Risk%20Analysis%20and%20evaluation.

5 The list of failure modes is available at: https://github.com/lebriag/TAI-PRM/ tree/main/Support/Failure%20modes.

6 The ranking information is available at: https://github.com/lebriag/TAI-PRM/ tree/main/Support/RPN%20ranking%20tables.

7 Tables available at: https://github.com/lebriag/TAI-PRM/tree/main/Support/ RPN%20ranking%20tables.

8 Example result available in Heat Map Construction.pdf at: https://github.com/ lebriag/TAI-PRM/tree/main/Support/

9 https://futurium.ec.europa.eu/en/european-ai-alliance/pages/welcome-altai-portal

10 https://www.msci.com/our-solutions/esg-investing/esg-ratings

11 An extension of required information is available at: https://github.com/lebriag/TAI-PRM/ tree/main/Support/Failure%20modes.
