# Supplementary material for: TAI-PRM: trustworthy AI—project risk management framework towards Industry 5.0
Source: AI Ethics. 2024 Feb 14;5(2):819–39. doi: 10.1007/s43681-023-00417-y (PMC12058918; doi:10.1007/s43681-023-00417-y)
Supplement: Supplementary file 1 — Supplementary file1 (DOCX 23 KB) [file 43681_2023_417_MOESM1_ESM.docx]

Supplementary Material – ASSISTANT case study

Table 1: Process Planning artefacts (PP)

| Process | Artifact | Definition/functionality | AI Taxonomy [[1]](#_bookmark0) |
| --- | --- | --- | --- |
| Manager | Chatbot | The chatbot can interact with the user by  providing information about process plans. | Communication / Natural language |
|  | Data Storage | A Mongo database to store data in the short  term | Services |
|  | Graph  Visualisation | The visualization of the graphs which allows  user interaction | Services |
|  | User Interface | A user interface provides the central interface  to various users. Different roles in production after authentication. | Services |
|  | Design model visu-  alisation | The user interface via suitable visualization  options or interfaces to simulation software or three-dimensional visualization software. | Services |
| Designer | Product analysis | A resource-neutral precedence graph de-  scribing the individual resource-independent tasks for assembly. | Planning / Searching |
|  | Production system analysis | Extracts a model of the production system  using the three-dimensional production sys- tem representation. | Planning / Searching |
|  | Production process assignment | The process graph as output provides, for  each necessary process step, all possible re- sources and tools that can fulfil the respec- tive product and process requirements | Planning / Searching |
|  | Process monitoring assignment | Enhances the resulting process graph by all  possible resources to perform the required process monitoring steps. | Planning / Searching |
| Predictor | ML model | In the case of high data availability, machine  learning models are trained with historical process plan data. | Learning / ML |
|  | Fuzzy inference | In the case of low data availability, fuzzy logic  is being used to model the relationship be- tween the features of process plans and re- sulting KPIs | Reasoning / Common Sense Reasoning |
| Optimizer | Selection model | The optimizer selects the optimal process  plan concerning the KPIs predicted (Process predictor) | Planning / Optimisation |
|  | Multibody simulation | The assembly plan is applied to an existing  simulation model of the production system that executes the assembly automatically in a virtual manner. | Services |

Table 2: Production planning and scheduling artefacts (PPS)

| Artifact | Definition/functionality | AI Taxonomy [[1]](#_bookmark0) |
| --- | --- | --- |
| User interface | The main functionalities are: Retrieving a production  scenario, setting targets and constraints, retrieving pre- viously calculated planning decisions to be used in the simulation, running the simulation, and comparing re- sults. | Services |
| Simulation client | The simulation client consists of a programmatic wrap-  per and an integrated material flow simulation tool. | Services |
| Analytics | Planning and scheduling tools and their machine learn-  ing models are summarized as Analytics. | Planning / Planning  and Scheduling |
| Domain Model and Extension | The domain model is intended to contain all the data rel-  evant for production planning and production schedul- ing in a production system. | Services |

Table 3: Real-time control and actuation artefacts (RTC)

| Artifact | Definition/functionality | AI Taxonomy [[1]](#_bookmark0) |
| --- | --- | --- |
| Execution control and Reconfiguration | Execution Control and Reconfiguration adjusts the pro-  duction process based on data from the Digital Twin. It is the general controller for real-time production. The quality control is done as the combination of historical and streamed statistical analysis and machine learning methods to evaluate and predict the state of the process (machinery and equipment conditions) and the state of the products. | Services & Learning / ML |
| Digital Twin of Execution | This module (DTE) interacts directly with the real  world. More specifically, the DTE consumes informa- tion about the workstation area layout, the resources (robot operations, robot state), and the different parts (consumables, assembly parts, fixtures) that exist in the real world. | Learning / ML & Planning / Planning and Scheduling |
| Human body detection and human task prediction | The detection of human presence is a mandatory compo-  nent in an H-R collaborative assembly system for ensur- ing the safety of the operator. The robot follows a pre- defined trajectory to execute its task. The Human Task Prediction (HTP) component extracts important infor- mation for improving the worker’s collaboration with the system. | Perception/ Com-  puter Vision & Integration and In- teraction / Robotic and automation & Learning / ML |
| Interfaces | Dedicated interfaces on smart devices for the Production  Manager to check the execution status as coordinated by the AI-based controller and to evaluate recovery plans generated by the controller. | Perception / Audio Processing & Services |
