# Supplementary material for: TAI-PRM: trustworthy AI—project risk management framework towards Industry 5.0
Source: AI Ethics. 2024 Feb 14;5(2):819–39. doi: 10.1007/s43681-023-00417-y (PMC12058918; doi:10.1007/s43681-023-00417-y)
Supplement: Supplementary file 2 — Supplementary file2 (DOCX 36 KB) [file 43681_2023_417_MOESM2_ESM.docx]

Supplementary Material – Risk Classification

# Nomenclature for Metrics 1

Table 1: Metrics Nomenclature

| Symbol | Name | Comments |
| --- | --- | --- |
| *β* | Failure effect probability | Conditional probability related to the sever-  ity of a failure effect. |
| *α* | Failure mode ratio | Ratio of the failure mode with respect to the  overall failure modes that can occur on a se- lected AI asset. |
| *γ* | Failure rate | Failing frequency for an AI asset, expressed  in units of time or operational cycles |
| *C_m_* | Failure mode Criticality Number | Metric to classify as a combination of prob-  abilistic and temporal risk effects, estimated as the multiplication of *γβαt* |
| *C_r_* | Criticality number | Total probabilistic and temporal risk effects  of the AI asset. Calculated as the sum of the *C_m_* overall the failure modes of the same component |
| *t* | Time/number of activities | Time or cycles in which the AI asset has been  used. It must be in the same units as the *γ* metric. Different sub-indexes are used to define the referencing time (e.g. *t_maintenance_*) |

# Severity Classification 2

A severity classification is helpful to provide a qualitative measure of the 3

potential resulting consequences from a failing item. Therefore, a severity 4

classification should be assigned for each failure mode and analysed compo- 5

nent. If no categories can be defined, a similarity with loss statements based 6

upon loss of system inputs or outputs shall be developed and included within 7

the FMEA/FMECA ground rules. A multi-failure description should deter- 8

mine the severity level that considers each ethical-based FMECA issue. The 9

table shows a proposition based on considerations of ethical and security- 10

based concerns. This table will feed the critical matrix by setting severity 11

categories with a critical number. 12

The severity code imposes the definition of low, minor, and significant 13

injury. These levels imply: (1) Low-level exposure: An exposure at less 14

than 25% of published Threshold Limit Value (TLV) or Short Term Expo- 15

sure Limit (STEL). (2) Minor Injury: A slight burn, light electrical shock, 16

minor cut or pinch. First aid can handle these and are not available to be 17

recorded following OSHA or considered as lost time cases. (3) Significant 18

19 Injury: Requires medical attention other than first aid. This is a medical

20 risk condition.

21 Prioritization is given to the system based on the ES&H scale. If the

22 levels based on this scale are acceptable under the institution’s risk appetite,

23 the severity ranking based on customer satisfaction could be used next.

## Severity Classification - Based on System Impact 24

Table 2: Severity Classification Based on System Impact

| Severity Designation of the Failure mode and Its Effect  Description | Severity  Rank | Failure Severity  Classification |
| --- | --- | --- |
| Failure would cause loss of life or total disability to personnel, Failure would cause identifiable catastrophic damage to the system and repairs that are beyond the capability of the user or contractor to resolve the effects, Failure would lead to violating any regulatory consider- ation set as fundamental rights, Failure would lead to violating principles that cause a non-recoverable and un-  dermining of the users and environmental well-being | 10 | Catastrophic (A) |
| Failure would cause severe disabling injury or severe occupational illness to personnel, Failure would cause identifiable critical damage to the system and extensive repairs to resolve the effects, Failure would lead to vi- olating principles that cause severe undermining of the users, and environmental well-being, Failure that can  cause fire or environmentally adverse conditions. | 8-9 | Critical (B) |
| Failure would cause a minor injury or minor occupa- tional illness to personnel that may require hospitalisa- tion, but failure is not disabling, Failure would cause identifiably marginal damage to the system an accept- able level of repairs and downtime to resolve effects, Failure would violate principles that will undermine the users and environmental well-being that could be man- aged with proper implementation actions, The severity level is high and activates alarms, safeguards, and re- quirements of special system attention, Can cause con-  trollable environmentally adverse conditions. | 6-7 | Marginal (C) |
| Failure would cause minor injury to personnel, but those injuries would not require hospitalisation, or failure would cause minor occupational illness, Failure would cause identifiable minor damage to the system and mi- nor repairs and short downtime to resolve effects, Fail- ure would lead to violating principles that cause minor undermining of the users and environmental wellbeing, The severity level activates alarms, safeguards, and re-  quirements of special system attention. | 3-5 | Minor (D) |
| Failure would cause less than minor injury and no occu- pational illness, Failure would cause negligible damage to the system and insignificant or no downtime to re- solve effects, Failure is not credible., There is no impact  on the environment. | 1-2 | Negligible |

## 25 Severity Classification - based on user satisfaction

Table 3: Severity Classification based on user satisfaction

| Severity Designation of the Failure mode and Its Effect  Description | Severity  Rank | Failure Severity  Classification |
| --- | --- | --- |
| Failure will result in significant customer dissatisfaction and cause non-system operation or non-compliance with  government regulations | 10 | Catastrophic (A) |
| Failure will result in a high degree of customer dissatis-  faction and cause non-system functionality | 8-9 | Critical (B) |
| Failure will result in customer dissatisfaction, annoyance  and deterioration of part or system performance | 6-7 | Marginal (C) |
| Failure will result in slight customer annoyance and  slight deterioration of part or system performance | 3-5 | Minor (D) |
| Failure is of such minor nature that the customer will  not detect the failure | <3 | Negligible (E) |

# Likelihood/Ocurrence Classification 26

A likelihood or occurrence ranking metric helps measure how frequently 27

an analysed failure mode could occur. The probability of occurrence (Pf) 28

should be based on the failure mode’s probability of occurring during oper- 29

ation time. The time frame’s homogenisation should be based on an hourly 30

or a 1E-6 hourly base for each failure mode considered. This designation of 31

time frame is used since its commonly used also in criticality analyses. 32

Table 4: Occurrence Ranking Criteria in function of temporal probabilities

d

| Severity Designation of the Failure mode and Its Effect  Description | Likelihoo  Rank | Failure Severity  Classification |
| --- | --- | --- |
| Once a week. High probability is defined as a single Pf  > 0.20 of the overall probability of failure during the item operating interval. | 10 | High probability (A) |
| Once every two weeks. Probability is defined as a single Pf > 0.10 but Pf < 0.20 of the overall probability of  failure during the item operating time interval | 7-9 | Probable (B) |
| Once a month. Occasional is defined as a single Pf >  0.01 but Pf < 0.10 of the overall probability of failure during the item operating time interval | 4-6 | Occasional (C) |
| Once every two months. Remote is defined as a single Pf  > 0.001 but < 0.01 of the overall probability of failure during the item operating time interval. | 2-3 | Remote (D) |
| An unlikely probability of occurrence during the item operating time interval. Unlikely is defined as a single Pf < 0.001 of the overall probability of failure during  the item operating time interval. | 1 | Unlike (E) |

Table 5: Occurrence Ranking Criteria in function of ratios

| Classification of risk | Ratio | Classification |
| --- | --- | --- |
| Very High | 1 in 2 | 10 |
| Very High | 1 in 8 | 9 |
| High | 1 in 20 | 8 |
| High | 1 in 40 | 7 |
| Moderate | 1 in 80 | 6 |
| Moderate | 1 in 400 | 5 |
| Moderate | 1 in 1,000 | 4 |
| Low | 1 in 4,000 | 3 |
| Low | 1 in 20,000 | 2 |
| Remote | less tan 1 in  10^6^ | 1 |

# Detection Classification 33

The following tables cover the detection classification ranking. As ob- 34 served, there are two tables in its use. The first defines the detection capac- 35 ity of failing conditions based on the products (e.g. inspection of products), 36

while the second is based on the detection of system control failures. 37

Table 6: Detection Ranking Criteria in function of temporal probabilities

| Detection Description | Detection  Rank | Detection Classi-  fication |
| --- | --- | --- |
| Very low (or zero) probability that the defect will be de- tected. Verification and controls will not or cannot detect  the existence of a deficiency or defect | 10 | Very Low (A) |
| Low probability that the defect will be detected. Verifica- tion and controls are not likely to detect the existence of a  deficiency or defect. | 8-9 | Low (B) |
| Moderate probability that the defect will be detected. Ver- ification and controls are likely to detect the existence of a  deficiency or defect | 5-7 | Moderate (C) |
| High probability that the defect will be detected. Verifica- tion and controls have a good chance of detecting the exis-  tence of a deficiency or defect. | 3-4 | High (D) |
| Very high probability that the defect will be detected. Verifi- cation and controls will almost certainly detect the existence  of a deficiency or defect. | 1-1 | Very High (E) |

Table 7: Detection Ranking Criteria in function of ratios

| Detection Description | Detection  Rank |
| --- | --- |
| Design Control will almost certainly detect a potential  cause/mechanism and subsequent failure mode | 1 |
| Very high chance the design control will detect a potential  cause/mechanism and subsequent failure mode | 2 |
| High chance ... | 3 |
| Moderately High chance ... | 4 |
| Moderately chance... | 5 |
| Low Chance | 6 |
| Very Low Chance | 7 |
| Remote Chance ... | 8 |
| Very remote Chance... | 9 |
| Absolutely Uncertain Chance ... | 10 |
