# Supplementary material for: TAI-PRM: trustworthy AI—project risk management framework towards Industry 5.0
Source: AI Ethics. 2024 Feb 14;5(2):819–39. doi: 10.1007/s43681-023-00417-y (PMC12058918; doi:10.1007/s43681-023-00417-y)
Supplement: Supplementary file 3 — Supplementary file3 (DOCX 44 KB) [file 43681_2023_417_MOESM3_ESM.docx]

Supplementary Material - Metrics

1. **Metrics** 1

The TAI-RPM introduces a set of metrics to correlate KPIs to specific 2

cases and products. The indicators obtained from the metrics will accredit 3

users to manage and act on the system based on risk-related information. 4

Furthermore, the proposed metrics are associated to system states to pro- 5

vide understanding on the level of improvement, risk state, and performance 6

blended with ethical considerations. Specifically, four categories of metrics 7

are proposed for the manufacturing sector on the processes associated to 8

TAI-RMP. 9

Table 1 includes the reference nomenclature used for the overall metrics 10

description included in next Subsections. 11

Table 1: Metrics Nomenclature

| Symbol | Name | Comments |
| --- | --- | --- |
| *β* | Failure effect probability | Conditional probability related to the sever-  ity of a failure effect. |
| *α* | Failure mode ratio | Ratio of the failure mode with respect of the  overall failure modes that can occur on a se- lected AI artifact. |
| *γ* | Failure rate | Failing frequency for an AI artifact, ex-  pressed in units of time or operational cycles |
| *C_m_* | Failure mode Criticality Number | Metric to classify as combination of proba-  bilistic and temporal risk effects, estimated as the multiplication of *γβαt* |
| *C_r_* | Criticality number | Total probabilistic and temporal risk effects  of the AI artifact. Calculated as the sum of the *C_m_* over all he failure modes of the same component |
| *t* | Time / number of activities | Time or cycles in which the AI artifact has  been used. It must be in same units as *γ* metric. Different sub-indexes are used to de- fine the referencing time (e.g. *t_maintenance_*) |

- 1. *Metrics for FMEA and FMECA* 12

When the user will perform any of the analyses using FMEA or FMECA 13

then these metrics can be used to trace the risk tendency. The metrics should 14

be grouped by specific risks on each trustworthy requirement. 15

When the information for failure rates associated to each of these groups 16

(*γ*) is available, the criticality number (*C_r_*) estimations must be used for this 17

propose. From these estimations, a criticality matrix should be constructed 18

19 to facilitate its tracking and visualization during subsequent stages of de-

20 velopment. When there is no information available for *γ*, the risk priority

21 number – RPN – can be used as a replacement to create the KPIs associated

22 to this metric.

23 For example, and considering *γ* is available, the Criticality Number is

24 used as accumulated critical value over the same AI artifact and same e-risk

25 (Human Agency and Oversight or Accountability). The same scale must be

26 used if several possible sources of critical numbers are used for comparison

27 of intrinsic risk level – *j* in the next formula: *C_r_* = Σ*j*

*n*=1

*cm,i*

28 The Table 2 describes the metrics associated to FMEA and FMECA:

Table 2: FMEA and FMECA metrics

| Name | Definition |
| --- | --- |
| Ethical Critical  Number (ECN) | Criticality number for an specific trustworthy requirement and AI arti- fact. *ECN* = Σ*k c_r,i_* where k identifies the requirement.  *n*=1 |
| Ethical Relative  Criticality Number (ERCN) | Ratio of item criticality number an specific trustworthy requirement  and AI artifact over the total critical numbers produced by the system:  *ERCN* = Σ *kc_r,i_/* Σ *jc_r,i_* Where *k* is previously defined and *j* is  *n*=1 *n*=1  the scale. |

29 *1.2. Framework general and ethical based metrics*

30 These metrics defined in this Subsection support the general evaluation

31 of the Risk Management Process. They are based on the general findings of

32 the TAI-PRM. These are complementary to the decision-making processes of

33 the risk status on an AI artifact under evaluation. Most of the KPIs should

34 be associated to these metrics that represent the ratio values based on the

35 risk limits – the concrete risk appetite allocations within the heatmap and

36 the 4T’s.

Table 3: High level metrics for management of risk ratio at overall system level

| Name | Definition |
| --- | --- |
| %*_LU_* - Unacceptable likelihood risk ratio | The likelihood of risks to materialise with an intrinsic value higher than  the chosen by the management. It is calculated as: %*_LU_* = *N_i>LU_ /N_risk_* where *N_i>LU_* is the number of risk with likelihood over the stated limits and *N_risk_* is the failure modes identified. |
| %*_LA_* - Acceptance likelihood risk ratio | 1 – %*_LU_* |
| %*_SU_* - Unacceptable severity risk ratio | Number of risks with severity over limit chosen by the management Num-  ber. It is calculated as: %*_SU_* = *N_i>SU_ /N_risk_* where *N_i>SU_* is the number of risk with severity over the stated limits and *N_risk_* is previously defined. |
| %*_SA_* - Acceptable severity risk ratio | 1 - %*_SU_* |
| *DC* - Detection Capacity | Average detection capacity of failure modes with risk levels over those in  the stated limits. This n umber describes the average on how the system fail. It is calculated as: *DC* = Σ*k RPN_i>DU_ /N_risk_* where *RPN_i>DU_* is  *i*=1  the risk priority number with detection over the stated limits, *k* are all  the risk over the stated limit and *N_risk_* is previously defined. |

- 1. *Independent to TAI-PRM* 37

These metrics are proposed to to track the AI artifacts status to to the 38

likelihood of failure events. These metrics are proposed to to track the AI 39

artifacts status with KPIs related to Computer Science specifics. The ones 40

suggested in this subsection are the most representative ones: availability, 41

capacity, performance, and the accuracy. However, these can be extended 42

depending on the AI artifacts – AI related or not. 43

Table 4: Metrics for AI components for software engineering non ethical based

| Name | Definition |
| --- | --- |
| *AIEC*  AI Effective Capacity | This metric represents the AI artifact up-time from deployment (not train-  ing if applicable). If the use of the AI artifact is discrete it refers to the number of uses. When it a DSS it must be considered the outcomes accepted by users with respect to the times it run. It is calculated as: *t_used_/t_total_* for discrete AI utilisation; *t_up−time_* for continuous time. |
| *AIPPM*  AI Planned Maintenance | This metric represents the ratio of time or cycles used for scheduled down-  time operations. It includes training /parametrization and AI maintenance. This is calculated as: *t_scheduled_/t_total_* |
| *AIDR*  AI Downtime Rate | This metric represents the ratio of unscheduled downtime. It must consider  the unexpected events when the AI was idle or offline. This metric provides insights on AI stability. This is calculated as: *t_unscheduled_/t_total_* |
| *AICU*  AI Capacity Utilization | This metric provides the amount of time when the AI should be utilised  with respect to the total available. The metric estimation is similar to *AIEC* with the difference that it considers only the functional time. It is calculated as: *t_used_/*(*t_total_ − t_scheduled_*) for discrete AI utilisation; *t_up−time_/*(*t_total_ − t_scheduled_*) for continuous time. |
| *AICI*  AI correction indicator | This metric provides insights on the efficiency of the AI artifact with re-  spect of a KPI (*KPI*) to perform contingency actions before they occur, compared to previous information available on the system. It is calculated as: (*KPI_initial_ − KPI_new_*)*/*(*KPI_initial_ − KPI_old_*) |
| *TP*  True Positives | A classification of data performed by the AI artifact that provides a correct  outcome. This metric represents the accumulated true positives over a period of time. |
| *TN*  True Negatives | A classification of data performed by the AI artifact that provides an out-  come that is not incorrect. This metric represents the accumulated true negatives over a time period. |
| *FP*  False Positive | A classification of data performed by the AI artifact that provides an out-  come that is not correct discarding the item from the right cluster. This metric is the accumulated false positives over a period of time. |
| *FN*  False Negative | A classification of data that provides an outcome not correct adding the item  into a category that is not adequate cluster. It represents the accumulated false negatives over a period of time. |
| *AI_A_*  Accuracy rate | Correct estimation as result of all *TP* and *TN* from the accumulated  classification operations of the AI artifact. It is calculated as: *AI_A_* = (*TP* + *TN* )*/*(*TP* + *TN* + *FP* + *FN* ) |
| *AI_M_*  Error rate | Incorrect estimation as result of all *FP* and *FN* from the accumulated  classification operations of the AI artifact. It is calculated as: *AI_M_* = (*FP* + *FN* )*/*(*FP* + *FN* + *FP* + *FN* ) |
| *AI_P_*  Precision rate | This metric provide the frequency of positive estimations. It is calculated  as: *AI_P_* = *TP/*(*TP* + *FP* ) |
| *F* 1 *− score* | This metric provides a mechanism to measure the AI classification per-  formance for predictors. It is calculated replacing the true negative by true positive to avoid the negative factors. The equation is: *AI_F_*_1_ = 2*TP/*(2*TP* + *FP* + *FN* ) |

- 1. *Environmental, social, and governance metrics* 44

Environmental, Social, and Governance (ESG) is a broad field with many 45

different investment approaches addressing various investment objectives that 46

cover three areas. The first is the ESG integration, which improves the risk- 47 return characteristics of investment. The second is the values-based invest- 48 ing, in which the investor seeks to align his investment with his norms and 49

beliefs. Finally, impact investing seeks to trigger changes on the social or 50

environmental scope. 51

A Morgan Stanley Capital International ESG Rating is designed to mea- 52

sure a company’s resilience to long-term, industry material, environmental, 53

social and governance (ESG) risks 1. They propose a rules-based methodol- 54

ogy to identify industry leaders and laggards according to their exposure to 55

ESG risks and the efficiency on the management of those risks by peers. 56

They also rate equity and fixed income securities, loans, mutual funds, 57

ETFs and countries. 58

Although ESG ratings are not directly linked to AI, they are suitable for 59

manufacturing companies to reference their environmental social and gover- 60 nance status. However, the use of the ESG metrics is interesting for gover- 61 nance, but not usable to track internal risk management processes associated 62

to the AI artifacts. 63

1h[ttps://www.msci.com/our-solutions/esg-investing/esg-ratings](http://www.msci.com/our-solutions/esg-investing/esg-ratings)
